# P04.86. Socio-demographic variations in barriers to participation in an acupuncture clinical trial

**DOI:** 10.1186/1472-6882-12-S1-P356

**Published:** 2012-06-12

**Authors:** T Tan, S Xie, S Li, R Lam, J Farrar, J Mao

**Affiliations:** 1 University of Pennsylvania Health System, Philadelphia, USA

## Purpose

As breast cancer survivors (BCS) increasingly use complementary and alternative medicine, randomized controlled trials (RCT) are needed to assess the safety and efficacy of these therapies to guide appropriate clinical use. However, many RCTs face poor patient accrual, especially among populations at risk for health disparities. The purpose of this study is to quantify the barriers to participation in an acupuncture clinical trial among BCS, and to identify the socio-demographic factors associated with these barriers.

## Methods

We conducted a cross-sectional survey study at an outpatient oncology clinic in an urban academic hospital among post-menopausal women on adjuvant aromatase inhibitors for stage I to III breast cancer.

## Results

Of the 300 participants, 148 (49.8%) were willing to participate in an acupuncture clinical trial. Despite high interest, perceived barriers towards participation were common and included presence of placebo (45.9%), travel difficulty (45.6%), home responsibilities (45%), demanding job (35.6%), lack of interest in acupuncture (27.2%), and discomfort with experimentation (25.2%). Socio-demographic factors were significantly associated with these barriers. While white participants were more likely to consider travel difficulty a barrier, non-white participants were more likely to consider discomfort with experimentation a barrier (both p<0.05). Older participants were more likely to cite discomfort with experimentation and lack of interest in acupuncture as barriers, while younger participants were more likely to cite demanding job and home responsibilities as barriers (all p <0.05). In addition, women with lower education were more likely to report discomfort with experimentation, presence of placebo, and lack of interest in acupuncture as barriers (all p<0.05).

## Conclusion

Although nearly half of respondents reported willingness to participate in an acupuncture clinical trial, significant barriers towards participation exist and differ among populations. Future studies must address these barriers to ensure effective accrual and improve the representation of individuals from diverse backgrounds.

